# Handmade relief models as matters of concern: Maintaining, restoring, and repairing mountains?

**DOI:** 10.1177/03063127251346168

**Published:** 2025-06-14

**Authors:** Alain Müller

**Affiliations:** Institute of Cultural Anthropology and European Ethnology, Department of Social Sciences, University of Basel, Basel, Switzerland

**Keywords:** relief models, representation, practice, representational objects, maintenance, ontologies

## Abstract

Through a case study of what are known as ‘relief models’—for example of areas of landscapes—this article approaches representational objects *in* and *as* practice. Such an approach implies following the multiplicity of practices that are gathered in representational objects to bring them into and maintain their existence, especially those that often remain unacknowledged by analytical attention. Most discussions of representational objects focus on their representational capacities and properties, paying less attention to the activities that ensure their ontological security as objects. Those activities concern not only the manufacture of representational objects, but also their maintenance—which is placed at the heart of this discussion. The maintenance of relief models manifests itself as a semiotic-material ecology. Entangled here are the ontological tact of the craftsperson, the affordances, resistances, and responsiveness of the materials, and the meaning-makings and stories that articulate and guide maintenance and repair. The practice of maintaining such objects, however, diverges from their production. Their production essentially accommodates metric distance since representation involves transporting a ‘thing’ through chains of reference. On the contrary, their maintenance aims to accommodate multiple temporalities. This involves not only the ways of being in time that are specific to each material that composes the object but also the idealized past of an unused object, its worn present, and its anticipated (repaired) future. By playing with the double meaning of the word ‘representing’, this article speculatively questions the extent to which practices of maintenance of, and care for, representational objects can inform a *re-vision* and rethinking of the relationships to what they are meant to *re-present*—that is, to what counts as nature.

I work with this material all the time. They’re polyurethane resin sheets. So, it’s polyurethane resin with a filler inside. It’s very easy to work with it. It’s extremely stable in terms of humidity and heat. … When I make these things, the aim is for them to last. (Interview, Model craftsperson HL, translated from French by the author)

It is in these terms that HL, one of the last craftspeople in Switzerland to build realistic handmade archeological and topographical three-dimensional dioramas and models for museums—including the ‘handmade relief models’ that are of interest in this article—explains to me the importance of durability for the models he creates and the techniques he develops to this end. The craftsperson is concerned about the durability of handmade relief models, and hence their potential fragility. More generally, this involves the process by which these models take shape and evolve as material objects. This article examines the concern for making objects last, along with its consequent and symmetrical attention to fragility and temporality (Denis & Pontille, 2023; Domínguez Rubio, 2016).

This focus, however, contrasts with what matters most for the scientific ‘engagements’ and pedagogical ‘deployments’ of relief models (Burri & Dumit, 2017). These practices primarily focus on their representational capacity, often relegating the question of endurance over time to the background. According to expert literature that documents, defines, standardizes, and recursively informs their manufacturing and use in various practices, handmade relief models are three-dimensional, true-to-scale models that realistically, accurately, and mimetically represent specific landscape sections—especially mountains. This conventional definition also governs their dissemination and reception by non-expert publics, that admire, engage with, and experience them as stunningly mimetic copies of mountains. This therefore attests to the percolation of the dominant epistemological rhetoric of modern science into common sense, that is, the idea that scientific knowledge and representations are and ought to be truthful, objective, and neutral mirror-reflections of their objects (Müller, 2025).

Such a definition deploys its effects in a contingent and situated manner, but, indeed, it is itself situated in a specific historicity. Handmade relief models were mainly built between the 19th and 20th centuries but are still produced on an occasional basis to this day. They are part of the ongoing legacy of scientific three-dimensional models that have shaped the politics of the Enlightenment (Schaffer, 2004).

Handmade relief models have actively contributed to the invention of cartography as a scientific discipline. Perhaps even more so, they have been a material stumbling block to this invention. The construction of reliefs historically preceded the science of cartography and the making of maps (Hurni, 2006). The production of some topographic maps thus made use of preexisting models. This shows just how much cartographers had to rely on the taken-for-granted mimetic resemblance between a ‘real’ mountain and a model. Such a mimesis was expected to be so all encompassing that the model could serve as a surrogate for the real mountain in order to create a map of it.

Thus relief models have been crucial operators in the process of sectorization and objectification, contributing to the invention and control of ‘mountains’ and, more generally, of territory and of what counts as the distinct ontological domain of nature, of which cartography is co-constitutive (e.g. Pickles, 2003). Consequently, relief models have contributed to establishing the vision of a single, ontologically given ‘one-world world’, which cartography, and science in general, aims to accurately represent (Law, 2015). In particular, they are archetypal of the partition, typical of modern science, between two distinct ontological domains: that of an objective nature on the one hand, and its faithful representations on the other hand (Latour, 1999, p. 24). Numerous works have discussed such a binary partition that excludes any possibility of ontological relationality and multiplicity (e.g., Haraway, 1989; Latour, 1993; Mol, 2002).

Relief models also feature another legacy of scientific, three-dimensional models: They have been a ‘key medium of traffic between the sciences and the wider culture’, especially regarding pedagogy and popularization (de Chadarevian & Hopwood, 2004, p. 6). They have been, and at times still are, used as epistemic objects for educational and pedagogical purposes in geomorphology, archaeology, and other scientific practices, as well as by the military and the general public—especially in museums. In Switzerland, where I have been conducting my research, the craft of handmade relief models is considered a form of ‘tradition’. Here, relief models are cared for and maintained as heritage. The ALPS Swiss Alpine Museum in Bern houses the world’s largest collection of relief models, making it a testimony to this heritage. From this point of view, relief models have contributed not only to the invention of the mountains they are supposed to represent but also the invention of a nation-state, Switzerland, whose mountains and their representations indeed represent (i.e., stand for) national identity as imagined, depicted, articulated, and narrated.

Finally, handmade relief models are, and are expected to remain, analog—that is, non-digital. They have surprisingly resisted the increased digitization of contemporary scientific modelling—so much so that some professional and lay geographers and cartographers (e.g., Räber, 2006; Welter, 2013), and the craftspeople who make them (see e.g., Mair & Grieder, 2006), have defended their scientific and pedagogical usefulness and efficacy to this day. This is justified by several reasons. Unlike other types of three-dimensional scientific models, whose heuristic potential relies on haptic, tactile engagement (Griesemer, 2004), handmade relief models are primarily intended for visual engagement to minimize the risk of damage by repeated handling, as they are considered fragile. But this in no way reduces the heuristic potential of their analog three-dimensionality. Among the justifications cited for this heuristic efficacy, the most important is the relief models’ ability to ‘enable us to view a landscape from all sides in a truly vivid way in a very small space’ and to ‘both survey the whole and recognize the details’, in the words of Toni Mair (a Swiss craftsman known as one of the great model makers, deceased in 2015) and Susanne Grieder (a former curator at the Swiss Alpine Museum) (Mair & Grieder, 2006, p. 9, my translation). Therefore, this enables a form of immediate relationship and intimacy with the mountain through its representation, which, according to the two experts, is based on the ‘principle of human vision plasticity’. Relief models, in this view, and in light of modern science’s rhetoric, and the presupposition that vision is reason (Haraway, 1988; Keller, 2009), are considered the ultimate perfection of a true*-*to*-*nature rendering (Daston & Galison, 1992). Although digital techniques could be and sometimes are used to manufacture three-dimensional models (or parts of them), the result is, as I discuss more extensively elsewhere (Müller, 2025), often deemed unsatisfactory against the backdrop of these conventional expectations. This may be due to a lack of precision and accuracy needed to reproduce details—a level of detail that watchmaking and analog microtechnology have historically enabled and continue to enable today. This also means that handmade relief models, as their appellation suggests, are expected to be crafted individually, allowing them to be traced back to a specific creator. In turn, the creator’s reputation and skills contribute to the model’s value and heuristic efficacy. This is evidenced by the fact that the ALPS Swiss Alpine Museum preserves not only the relief models themselves but also the tools of specific craftspeople. This stands for and thus aims at preserving the legacy of their *savoir-faire*, such as the toolbox of the Swiss craftsman Hans Kappeler (1904–1984).

Therefore, just as models must be true to nature, they must also remain true to their authors, like modern art objects (Domínguez Rubio, 2016, p. 69). Notably, with the ‘enchantment of technology’ models can be like art objects, through a fascination for the technical skill involved in creating them (Gell, 1992). As I explore in the conclusion of this article, relief models can be viewed as hybrids of science and art, continuing the legacy of what Daston and Galison (1992) have shown in the case of 19th-century scientific paintings.

These features make relief models spectacular in the eyes of professional scientists and the general public alike. But for this effect to unfold in the foreground and for a mountain to be enacted in such a remarkable way, a series of behind-the-scenes supporting practices must take place. This article turns its analytical attention to these practices and activities, especially those aimed to ensure the admiration and the use of relief models as solid, consolidated objects, endowed with a given finitude and a more or less stable ontology—a potential on which their representational properties depend.

This article is structured as follows. First, I situate my discussion within the ‘turn to maintenance’. I then discuss my materials and methods, whose relationship I think of in a recursive heuristic, where materials call for methods which, in turn, produce new materials. Articulated in the light of this recursive heuristic, the main part consists of analytical descriptions of restoration and maintenance practices of historic relief models. Finally, I ask: To what extent can these practices inform a *re-visioning* and rethinking of the relationships to what the relief models are meant to *re-present*—that is, to what counts as nature?

## Turning the analytical attention advocated by the ‘turn to maintenance’ toward representational objects

By committing to this project, my analysis follows in the footsteps of the ‘turn to maintenance’ (Russell & Vinsel, 2018) and, more generally, of the analytical project of shedding light on the invisible, everyday work of maintenance, repair, and care for things (e.g., Denis & Pontille, 2015, 2019, 2022; Denis et al., 2015; Graham & Thrift, 2007; Jackson, 2014). The principles and ramifications of this field of research will be the conceptual and methodological guideposts of my analysis to ‘help … look in the right places for answers to questions’. (Hutchins, 1995, p. 287). The turn to maintenance starts from the observation that repair and maintenance activities are often institutionally invisible just as they often remain unacknowledged by analytical attention. My analysis sets out to further test and explore the analytical relevance and political implications of bringing them to light. In particular, analyzing repair and maintenance can serve as heuristic operators to study the complex relationships between humans and things (e.g., Denis & Pontille, 2022). This draws on and contributes to a material, ecological, and relational understanding of what constitutes the ‘social’ (e.g., Domínguez Rubio, 2016, 2020; Puig de la Bellacasa, 2016; Star, 1990, 1999; Star & Ruhleder, 1996).

It remains to be considered whether the practices of maintenance of realistically representational objects such as relief models present their own specificities. This question, which I will address in the next sections and revisit in the conclusion, arises in the context of a field where the majority of literature focuses on infrastructures and technical objects (see references above). A smaller segment of this literature, however, addresses art objects (Domínguez Rubio, 2016, 2020). This is of particular relevance to my analysis, as art objects share several features with relief models. These, in addition to features I discussed above, include their cyclical pattern of public display and storage in repositories.

On the other end of the spectrum, discussions on representational objects, namely scientific models, often focus on their ‘representational force’ from an epistemological standpoint (Suárez, 2004; also Knuuttila, 2011). Alternatively, they may address onto-epistemological questions related to representation as a practice from an STS perspective (e.g., Mol, 2002; Myers, 2015). However, these discussions typically do not explore the literal process of the *objectification* of representational objects, that is, the practices involved in maintaining them in existence as stable, durable, and consolidated *objects*.

## Materials and methods

My analysis adopts a ‘methodological sensitivity’ (Barla, 2021) that attunes ethnographic attention to craftspeople’s practices, their own ‘ecology of attention’ (Citton, 2016; see also Denis & Pontille, 2023), and the ‘ecology of materials’ (Bennett, 2010; Denis & Pontille, 2023; Ingold, 2012) in which this attention is embedded. To operationalize this methodological sensitivity, I approach handmade relief models through two main methodological principles: (1) examining them *in* and *as practice*, and (2) approaching them as processual *things* rather than stable *objects*. In what follows, I clarify what I mean by these two principles.

First, I propose to approach handmade relief models *in* and *as practice*. This aligns with the long-standing focus of STS on ‘practices’ (Coopmans et al., 2014; Latour & Woolgar, 1986; Lynch & Woolgar, 1990; Lynch, 1994; Pickering, 1995). It also resonates with scholarly discussions and debates about cartography and maps, which, as I have suggested, share common features with handmade relief models (see in particular Pickles, 2003). Such a similarity allows me to clarify both the contributions and the potential gaps that adopting this principle produces. Indeed, Kitchin and Dodge (2007) propose to suspend the view according to which ‘maps remain *secure* as spatial representations that say something about spatial relations in the world (or elsewhere)’, a view that keeps providing ‘ontological *security* for maps’ as a ‘coherent, stable product’ (p. 334, emphasis added). In contrast, they propose approaching maps as ‘of-the-moment, brought into being through practices (embodied, social, technical), remade every time they are engaged with’ (Kitchin & Dodge, 2007, p. 335). In this perspective, they argue, maps ‘are always mappings[,] spatial practices enacted to solve relational problems’ (Kitchin & Dodge, 2007, p. 335).

Accordingly, my analytical stance does not aim to grant any a priori ‘ontological security’ to relief models. However, I do argue that applying Kitchin’s and Dodge’s proposal to the study of relief models risks ignoring the efforts and endeavors of the craftspeople who work on building the models and making them last, as expressed by HL in my epigraph. Consequently, this contributes to the invisibility of a whole field of activities and practices aimed at making, keeping, and maintaining models as solid and durable objects with well-defined finitude, shape, and properties. Such a great effort is not in vain: Made of plaster with wooden and/or resin elements, handmade relief models come in a variety of sizes and compositions. However, the typical model fits into 1 square-meter, is around 50 centimeters high, and weighs several dozen kilograms. Such a presence in the world does not evaporate when the models are not engaged in and as practice. This is also a consequence of the fact that the efforts to consolidate and keep relief models ontologically stable are not the sole responsibility of human agency. Once stabilized as objects, the efforts are delegated to the agencies, capacities, and properties of the used materials which hold everything together, that is, partake in securing the ontological solidity of the models. In other words, the stable ontology of the models requires an active contribution of plaster, wood, ceramics, glue, and resin.

This does not mean, however, that their ontological security is guaranteed once and for all. Each material of these complex assemblages has its own properties and temporalities (Bensaude-Vincent, 2022; Domínguez Rubio, 2016), which makes them subject to degradation, instability, and fragility. Hence, models remain fragile and vulnerable to the wear and tear of time and handling. Their durability therefore requires conservation, meaning a wide range of maintenance, repair, and restoration activities and practices to maintain them as they ought to be. Therefore, among the many practices and activities on which relief models depend and to which they give rise, some, in fact, strive to solve a problem that is neither representational nor spatial, but material: holding and maintaining plaster, wood, and ceramics together and ensuring that the resulting material assemblage stands the test of time. From this perspective, ontological security should be approached as a practical accomplishment, as the provisional result of continuous activities, that is, as ontological *securitization*. Such an invisible work of ‘minor acts’ (Domínguez Rubio, 2016), as Star (1999) argues, demands to be ‘surfaced’.

Relief models make this dimension visible, literally ‘into relief’—unlike maps, which exist as drawings or prints on paper or, more commonly today, as digital interfaces. These interfaces and the digital infrastructure on which they depend (for a discussion see Rankin, 2016) also require massive maintenance, but this work is extensively externalized and distributed, rendering it almost invisible (Star, 1990). In fact, its invisibility is so pronounced that it remains unacknowledged by analyses examining maps in and as practice. Relief models are interesting heuristic entry points for analyzing the maintenance of representational objects, as they locally concentrate the practices and activities required to keep them in existence, and thus render them locally analyzable.

Now, drawing on Latour’s (2004) distinction between ‘matters of fact’ and ‘matters of concern’, as well as Ingold’s (2007) distinction between ‘materiality’ and ‘materials’, Denis and Pontille argue that the term ‘object’ conveys a finite, stable dimension, ‘a matter of the self-evident and of the “already there”’. However, ‘thing’ evokes ‘what is not self-evident, what can disappear, dissolve, crumble, and which requires constant actualization’ (Denis & Pontille, 2022, p. 21, my translations). This distinction is also subject to a political dimension, a ‘material politics’ (Gregson, 2011), since the passivation of materials, that is, their semiotic relegation and material transformation into passive objects is at the heart of the modernization process and its characteristic modes of domination (see Bensaude-Vincent, 2022).

This brings me to the second methodological principle, which approaches handmade relief models both as ‘things’ and as matters of concern. A double dimension unfolds here. The first one, following the discussion above, implies approaching relief models as ‘things’ that are ‘constantly falling out of place’ rather than stable objects (Domínguez Rubio, 2016, p. 60; see also Denis & Pontille, 2022). This implies extending the analytical attention beyond the sole isolated artifacts, which are often understood as ‘passive containers of external social forces’ (Denis & Pontille, 2015, p. 14) to reach the ‘stuff that things are made of’ (Ingold, 2007, p. 1, cited in Denis & Pontille, 2015, p. 13), and therefore to ‘follow the materials’ (Ingold, 2007), and to approach ‘things’ as ecologies of materials. Further, one should not forget that relief models, once maintained—albeit temporarily and provisionally—remain more or less secured in a stable state, what Domínguez Rubio refers to as an ‘object-position’. This is a state ‘to which … things are subsumed in order to participate in different regimes of value and meaning’ (Domínguez Rubio, 2016, pp. 61–62) and engaged with as ‘working’ objects—in the case of relief models as working representational objects. From this perspective, my analysis aligns with Domínguez Rubio’s proposal to ‘not locate our enquiry at the level of objects (i.e., positions) or at the level of things (i.e., material processes) but rather in that space lying betwixt and between objects and things in which much of our lives take place’ (Domínguez Rubio, 2016, p. 64).

Following the sociomaterial histories of the materials involved in the fabrication of relief models—an approach like Angus’ (2024) rewriting of photography’s history through its constituent materials, or Mareis’ (2024) examination of rubber’s history (see also Bensaude-Vincent, 2022)—could situate these models within a global sociomaterial history of modernity. Such an analysis would reveal an interesting contrast: The invention and depiction of an idealized and objectified nature, ontologically purified from human activities—a project that was historically actively defended by relief makers and in which relief models have been and continue to be active operators—required resource extraction from nature to produce the wood, plaster, and resin necessary for their manufacture.

The second dimension relates to the very etymology of the word ‘thing’. ‘Thing’ stems from Old English ‘þing,’ which refers to ‘meeting, assembly, council, discussion,’ and later ‘entity, being, matter’ (Online Etymology Dictionary, 2025). As Latour (2004, p. 233) notes, ‘a thing is, in one sense, an object out there and, in another sense, an *issue* very much *in* there, at any rate, a *gathering*’. From this point of view, approaching things as such also means seeking to ‘detect how many participants are gathered in a thing to make it exist and to maintain its existence’ (Latour, 2004, p. 246). Indeed, multiple practices such as geomorphology, cartography, craft, museography, conservation, and archeology are gathered in relief models to make them exist and to maintain their existence. In this perspective, the existence of relief models depends precisely on what Stengers (2005) has defined as an ‘ecology of practices’.

Consequently, the analysis presented here, while locally situated backstage to this ecology of practices, is nevertheless grounded in a wider inquiry that seeks to trace what I call, drawing on Bensaude-Vincent (2022), the ‘sociomaterial biographies’ of relief models. As discussed by Bensaude-Vincent (see also Daston, 2000), the notion of biography is particularly enlightening in terms of the analysis I am considering here. The methodological heuristic of the use of the term ‘biography’ to refer to the ongoing transformations and becomings of ‘things’ lies mainly in its propensity to call special attention to their fragility, for these ‘are neither immutable nor stable’—they ‘come into being and … disappear’ (Bensaude-Vincent, 2022, p. xxv).

Following this logic, I here resume an inquiry I began elsewhere, tracing and recomposing the manufacture of relief models (Müller, 2025). The aims of this inquiry are twofold: (1) to trace these biographies across the broader networks and ecologies of practices that both bring and maintain relief models into existence; and, symmetrically, (2) to follow how relief models, once stabilized in their object-position, actively operate as boundary-objects to mediate the collaborations of these multiple practices; and, additionally, participate as ontological operators in defining what a mountain is and, more broadly, what counts as nature.

From a methodological standpoint, this broader inquiry relies on the constitution of a comprehensive corpus combining an analysis of specialized literature with a multi-sited, multi-scalar, and multi-temporal ethnography (Müller, 2016) of the situated practices involved in crafting, mediating, maintaining, and admiring relief models. The materials discussed in this article are integrated to and complemented by this broader corpus. At the same time, in order to provide thick descriptions of the situated practices at stake, they draw on analytical descriptions that are composed of and articulated through what Strathern (1999, p. 6) calls ‘the ethnographic moment’, that is, the interplay between ‘the understood (what is analysed at the moment of observation)’ and ‘the need to understand (what is observed at the moment of analysis)’. Here, the moment of observation was a day organized by the Swiss Alpine Museum for the restoration of historic relief models kept in its repositories. I attended it with the aim of paying particular attention to model maintenance and restoration practices, and to trace the ontological operations involved in the process. The analytical descriptions articulated on this basis focus on two particular situations. The first involves the restoration of a worn-down relief model. The second involves the repair of a relief model that got broken after it had been loaned to another museum.

## Maintaining and restoring relief models: An ethnographic journey into the underground world of matters of concern

Driven by my concern for the becomings of handmade relief models, I attended a conservation session at the Swiss Alpine Museum’s repository in Zollikofen, in the suburbs of Bern. It was the summer of 2023. It was a hot day, with the temperature hovering around 35°C. The museum’s collections manager welcomed me and accompanied me to the repository’s elevator, an airlock that took me from the ground floor to the fifth basement of a gigantic hangar, and from 35 to 18°C, as required for the conservation of museum objects.

Access to the practice I was interested in involved crossing a threshold that, from the perspective of the broader public, separates the visible from the invisible, the surface from the underground. Crossing that threshold took me from the world of taken-for-granted ‘objects’—matters of fact—to that of ‘things’ that are objects of care—matters of concern. As discussed earlier, this is not a substantialist distinction among different ontological states. Rather, it is relational and contingent, designating specific modes of relation and engagement. In other words, the objects of some practices and situations are the things of other practices and situations, and this distribution is at the heart of a political economy that organizes practices and activities around oppositions such as valued/devalued, visible/invisible, included/excluded, and internalized/externalized (see Puig de la Bellacasa, 2011; Star, 1990). In the case of relief models, and the museum’s policies in which they are embedded, this political economy is literally materialized by the design of the built environment. Objects—matters of fact—shine on the surface under spotlights in the city center museum. Things—matters of concern—are brought underground, cared for in the intimacy of the suburban repository where they are not displayed for the public.

As I crossed this threshold, I followed the relief models themselves, which had taken this elevator some time before me. They had to leave the surface, where they had been admired, used, and engaged with as representational objects, in order to be taken care of.

Elsewhere, I have described the efforts to accommodate and assemble the materials and the obligation of the practice, that is, the objective to mimetically, morphologically, and materially resemble the ‘real’ mountain that it ought to *re-present* (Müller, 2025). Once the relief models are built, they become representational objects in their own right. They can, as matters of fact, rely on a certain ontological security to circulate and act, that is, become full-fledged actors. This is where their biography begins.

However, as their sociomaterial biography unfolds, relief models are subjected to all manners of trials and tribulations. They are cracked by manipulation, transport, and the passage of time. For those in charge of preserving these things, and for the ethnographer caring to reconstruct their biographies by following their most intimate moments, they again become ‘matters of concern’ here and now. This overturning moment forms the entry point for the analysis that I unfold in the following ethnographic descriptions.

That day, two relief models were to be the subject of care by a professional restorer. He was not a specialist but had dealt with some of them in a previous mandate at the Swiss Alpine Museum.

### Accommodating and reassembling materials, objectives, and temporalities

**Figure 1.**
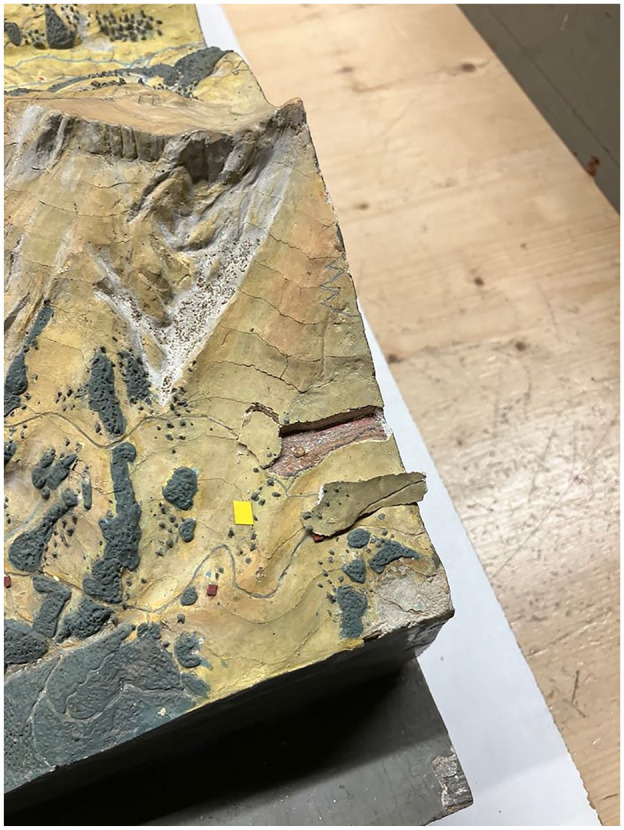
Post-it notes on relief model.

The first model to be restored had been found in a school attic and was entrusted to the museum. It showed signs of wear. Tiny Post-it notes over the cracks indicated where the restorer should intervene (Figure 1). These had been placed beforehand, in alignment with the conservation policy of the Swiss Alpine Museum. That policy reflects the general trend of favoring conservation over restoration, that is, intervening as little as possible, and not hiding the intervention.

This brings to light an important dimension that I revisit in the conclusion. While the realism of the original relief models consists of *re-presenting* a mountain as part of an idealized, perfect nature that remains untouched from human activity and requires conservation efforts to remain as such, current techniques for conserving museum objects advocate for another form of realism: the passage of time, no longer seeking to hide cracks and wear—a form of temporal realism.

In line with this policy, the goal of the intervention was to ensure that the relief model could retain its structural integrity by avoiding ‘substance loss’ (*Substanzverlust* in German): the Post-it notes indicated spots where there was a risk of this happening. Here, the notes materialized the session’s objective and marked its progress. They superimposed an objective, a story to come on the artifact and the marks of its past, in a movement in which the past and a prospective future were brought into presence through the mediation of those little squares of paper.

**Figure 2.**
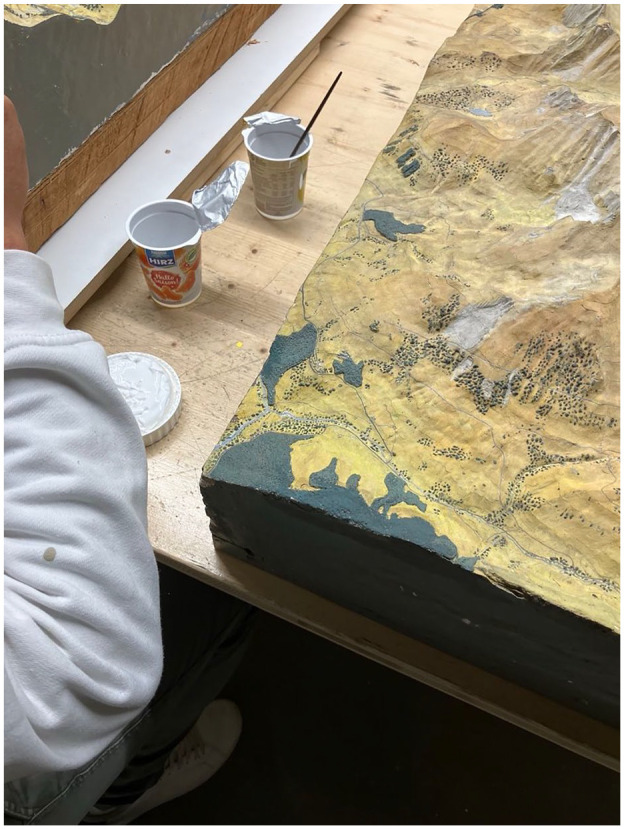
Preparation of the substance for intervention.

First, the restorer prepared the substance for his intervention, an acrylic base that he mixed in used yogurt cups. Then, the intervention began. The first movements were extremely meticulous (Figure 2).

The restorer demonstrated impressive concentration and control, and what—drawing on Despret (2021) and Denis and Pontille (2022)—I have elsewhere called ‘ontological tact’ (Müller, 2025). This is a particular attention, attunement, responsibility, and ‘responsiveness’ (Code, 1988) toward the actions and reactions of materials and the way they respond (Bensaude-Vincent, 2022). This tact was all the more important since, while the relief models ought to represent the mountain, they do so on a scale. This scaling gives the model its representational capacity by enabling it to render the immensity of the mountain in a reduced space. Nonetheless, it is often taken for granted or even neglected by the general public, which rather marvels at the fact that these models are mimetic copies of real mountains. Here, on the other hand, scaling took on its full meaning and consequences: the immensity of the represented mountain was reduced to minuscule areas of around one square millimeter.

Under these conditions of extreme attention to detail, one of the challenges, according to the craftsman, was what he called the ‘conflict between materials’. Indeed, the relief has the peculiarity of having a top layer of plaster over an underlay of wood. Each material responds differently to the passage of time, having its own ‘*manières d’être au temps*’ or ways of being in time (Bensaude-Vincent, 2021, p. 16), which results in cracks. To this extent, a relief model is, as an assemblage of materials, also a *polychrony* (p. 17)—an assemblage of various temporalities (see also Domínguez Rubio, 2016). Hence, the craftsman, his work, and the intervention of the resin he applies mediate between the materials and their temporalities. The action of the resin, deployed through the work of the restorer, involves accommodating and mediating between different materials, each with their own temporalities.

Handmade reliefs, however, are not only fragile ecologies of materials. As ‘things’ and matters of concern they are also ecologies of practices (Stengers, 2005). Stengers’ proposal to think in terms of ecologies of practices points to a twofold analytical project that allows to further multiply lines of analytic inquiry. First, it highlights the specificity of each practice. Since ‘no practice can be defined as any other, … approaching a practice then means approaching it as it diverges.’ (Stengers, 2005, p. 184) Second, it draws analytical attention to the ways in which different practices relate to each other and the ways in which ‘heterogeneous worldings [are] coming together as a political ecology of practices, negotiating their difficult being together in heterogeneity’, as summarized by Blaser and de la Cadena (2018, p. 4). In this perspective, handmade relief models, as matters of concern, are also political ecologies of the multiple practices that engage them.

Their existence is therefore always positioned at the interface of political ecologies of practices, and materials. From this perspective, recomposing their sociomaterial biography involves addressing the ‘stuff they are made of’ and the multiplicity of the ontological operations required to hold them together, which requires particular attention to the affordances of materiality and materials, as well as the meaning-makings, the ‘order-words’ (Stengers, 2007), and the stories that articulate, organize, and guide the various practices that they gather—practices that, in turn, maintain their existence.

Model restoration accommodates materialities with stories, objectives, temporalities, and worldings that are specific to human modernity. It conserves, preserves, and holds together the relief-model-as-an-object with its ontological stability, keeping it ‘alive’ as a witness to the past (as a historical object) and as a representation of what counts as nature (as a representational object). The restorer’s intervention therefore introduces a new material—an acrylic base with its own temporality—into the ecology and polychrony of materials. It also brings specific stories: the unfolding of a very human, modern temporality of short, linear time, and the consequent need to maintain and preserve the integrity of the objects that matter against this backdrop.

In keeping Stengers’ problematization of ecologies of practices as a heuristic guidepost, there remains the question of the specific ways through which the maintenance of relief models, as a specific practice, diverges from the multiplicity of practices that they gather. Here, for heuristic purposes, let me mobilize my previous description of models’ manufacture (Müller, 2025). This involves accommodating in practice a double obligation, according to the conceptual vocabulary of Stengers (2005). That is, the obligation imposed by the objective of the practice, which acts as an ‘order-word’ demanding that the mountain be represented objectively and realistically, and the obligation imposed by the objects used, in this case the materials, which deploy their own agencies, capacities, and resistances. In this perspective, manufacturing relief models pertains to a situated, material practice in which the craftspeople’s actions, attunement, responsiveness, and ontological tact, the objective to be achieved with its specific requirements, and the objects used to achieve it respond to each other in a material-semiotic ecology. Here, words and things, meaning and matter, and stories and materials emerge and entangle in situation, *in* and *as* practice.

As per my initial observations, both conservation and maintenance also accommodate a double obligation. One is imposed by the materials and their responsiveness. The other is imposed by a different order-word than the one subjecting the manufacture of the relief model. This order-word is the need to preserve the relief by aligning it with the requirements and expectations of the museum. The latter, in turn, are informed by a conservation cultural policy.

Moreover, one of the challenges of manufacturing relief models is to accommodate a multiplicity of spaces. In fact, the work involves transporting the mountain through chains of reference so that it can remain constant and be re-presented in another location and a restricted space (Müller, 2025). Manufacturing therefore involves accommodating metric distance. The challenge of maintaining relief models is to accommodate a plurality of temporalities: the ways of being in time of the different materials and the idealized past and anticipated futures crafted by the objective of restoration. To this extent, the semiotic-material ecology of maintenance recombines differently from that of manufacture. Each practice, and the trials of strength (Latour, 1988) that punctuate it, make new actors, new mediators, and new parasites emerge (Serres, 2007). These can reconfigure the complex, unstable, and shifting ecology of relations between meaning and matter, stories and materials, and humans and non-humans. The analysis of the second situation will help to clarify this dimension.

### Making the before, the after, and the materials co-respond

**Figure 3.**
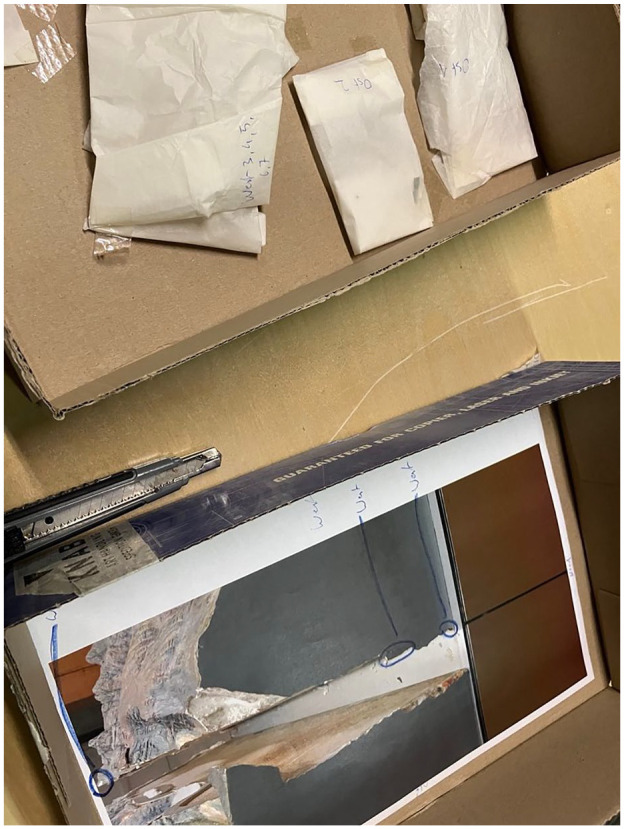
Photographs of the model taken after the accident.

A second relief model awaited the restorer’s intervention. The model had been loaned by the Swiss Alpine Museum for an exhibition in another European country, and suffered several points of damage during transportation. The relief model had the particularity of being composed of two platforms, each about one square meter in size. These had to be joined together for display but could be transported and stored independently, each in its own wooden crate. The corners and joints had been damaged in transit. The restorer was given small plastic bags containing the broken fragments and a list accompanying each bag. These elements indicated exactly where each fragment came from. Further, there were several photographs of the model that had been taken right after the accident. These also showed and cited the locations for each of the numerated fragments (see Figure 3). One bag, however, contained a few fragments labeled ‘unspecified’. Here again, the storyboard of the restoration-to-come had been pre-marked as a trail with small pebbles, yet leaving a small, unresolved part of the puzzle to the restorer’s ingenuity and capacity of solving it in practice (see Figure 3).

**Figure 4.**
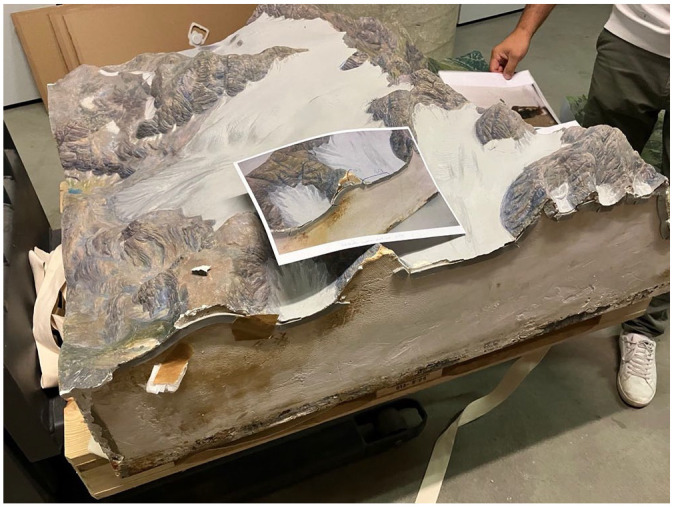
Photographs of the relief model in an earlier stage of its biography.

First, the labeled fragments were glued back together using an acrylic substance according to the pre-determined scenario. Then, the real puzzle began: Where did the unspecified fragments come from? The photographs of the relief model were used as a reference. A new actor in the situation emerged: the relief model in an earlier stage of its biography, before its accident, was brought back into the presence of the situation through the mediation of the photographs (see Figure 4). This implied a lot of to-and-fro between the photograph and the parts of the model showing cracks. Indeed, it was a real game of correspondence in which the search for solutions was not entirely satisfied, with two floating fragments remaining without any attributed cracks at the end of the process.

The term ‘co-respondence’ emphasizes the fact that while the main objective of model manufacturing consists in establishing a correspondence between the original mountain and its representation, the craftsperson actually accommodates this demand in a response-able partnership with the materials at hand—hence a co-respondence (Müller, 2025). The vignette I just unfolded shows that the work of repairing relief models also pertains to a form of co-respondence, where the main objective reconfigures the situation: It is no longer about matching the ‘real mountain’ and its modeled re-presentation, as in model manufacturing. Rather, it is a matter of matching the before and the after, that is, the re-presented past and the current state of the representational object. Once again, the objective of maintenance seems to lie in the accommodation of multiple temporalities—here, the (represented) object’s past, its present state, and an anticipated (repaired) future.

### Relief models as political ecologies of practices

Of course, maintenance remains maintenance with all that follows—that is, an externalized, invisible, and literally underground activity. It is always subject to what constitutes the main objective’s demands to bring and keep relief models primarily as representational objects, with their representational capacities, into existence—an additional demand to be accommodated in the practical maintenance situation. Once this and all other obligations of reparation are fulfilled, the model can be returned to its conservation case as a historical matter of fact. Or, it will be ready to be taken to the elevator again and display its full strength in re-presenting nature as a fact at the whim of exhibitions. In such a display, reified matters of fact re-present other reified matters of fact, temporarily obscuring the ‘webby’ and ‘thingy’ (Latour, 2004, p. 237) properties of relief models, mountains, and nature as matters of concern.

Thus, in order to be brought into and maintained in existence, relief models indeed require a collaboration between multiple practices, each with its objectives and interests. More generally, this pertains to a ‘sociomaterial alliance of multiple collaborations [where] humans … and materials … work together’, as Schürkmann (2022, p. 4) formulates it. Together, these allies must engage in scientific ‘interessement’ (Callon, 1984). This means that they must obey the order-word of modern science according to which relief models are and ought to be realistic and mimetic representations of natural landscapes. In other terms, the representational capacity of models constitutes the primary collaboration aim of the ecology of practices that they bring together. Its network of collaborations organizes around it, thus relegating other practices and their respective goals, notably maintenance, to ‘network externalities’ (Star, 1990).

## Conclusion: Speculating ways of relating to nature through chains of care

To conclude, I would like to raise two questions. The first concerns the lessons that can be drawn from the sociomaterial biography of relief models. As I have argued, these models emerge from and operate as boundary-objects in an ecology of practices. Such an ecology, in turn, shapes and engages the models with different objectives and interests. Hence, the required collaboration is always fragile and subject to constraints and negotiations. Engaging them as representational objects in order to solve representational and spatial problems seems to be at the forefront. However, a more invisible set of practices helps to achieve and maintain their ontological security as representational objects. This conclusion can undoubtedly be extended to all scientific practices. To grasp them as a collective activity, it is analytically fruitful to highlight all the practices and activities undertaken by what Becker (1974) calls the support personnel. Although this has found many reverberations in STS, notably through the work of Star (e.g. 1990, 1999) and the ‘turn to maintenance’, it has not been applied in its full scope to scientific practices and their objects. How are epistemic objects such as models maintained as *objects*? For whom are they matters of concern and care?

Griesemer (2004, p. 438) notes that ‘[3-D] models not only mediate between words and worlds … but also between … audiences and allies’. With this in mind, let us take a closer look at mediations between different practices. This raises a question that Griesemer (2004, p. 438) leaves hanging: ‘How … did mediation among audiences and allies come to depend on the representational functions of models?’ In other words, does the ecology of the practices that are gathered in relief models to make them exist and to maintain their existence, and the (non)recognition of model maintenance and restoration activities within this ecology, depend on the fact that they are representational objects? Is there a fundamental difference between maintaining and restoring a representational object and an object with a different mode of existence, for example, a piece of art such as the Mona Lisa, whose complex maintenance has been meticulously documented (Domínguez Rubio, 2016)?

Elsewhere, I have provided a fragment of an answer to this question by showing that geologists use old relief models to take stock of glacier melt (Müller, 2025). Relief models are therefore surrogates of not only the mountains they represent, but also of the mountains at the period of time they represented those mountains. So, to answer Griesemer’s question, the maintenance of relief models has to serve their primary goal, which is to represent the mountains and the territory as accurately as possible at a given moment in time. From this point of view, they are also historical witnesses, and their maintenance and conservation contribute to preserving and archiving the ‘mountains’ they ought to represent in their past state.

This brings me to my second concluding question, which concerns the possibility of imagining that the relationship of care to a representational object may have consequences for the modes of relationship to the reality it is supposed to represent. This is, in the case of models, the mountain and, more broadly, what counts as nature. Elsewhere, and as summarized above, I have discussed the trajectory that brings the mountain to its modeled re-presentation. This passage from matter to form relies on inscriptions and chains of reference that aim to keep ‘the mountain’ constant through transformations, and truth constant through chains of trust. Latour insists on the idea that what creates the robustness of chains of reference is that they can always be retraced. In other words, the passage from matter to form must be reversible and traversed in the opposite direction—step by step and inscription after inscription. Starting with the form, it must always be possible to return to the matter, ensuring that the represented object retains an unchanging, constant character throughout the transformations.

On the basis of this idea, I wish to raise the following speculative question by way of a concluding remark. The care given to representational objects—as I have unveiled in the case of handmade relief models—is a very specific mode of relationships between humans and things. Indeed, as it has been discussed within the framework of the turn to maintenance, maintenance practices can be analyzed as an act of taking care, especially in the light of the feminist politics of care (Fisher & Tronto, 1990; Murphy, 2015; Puig de la Bellacasa, 2011, 2017). This perspective contributes to political inquiry and speculation about the modes of relating to things and the world based on care and ‘response-ability’: ‘engag[ing] with the material-semiotic becoming of things’ (Puig de la Bellacasa, 2011, p. 96; see also Denis & Pontille, 2023; Hall & Smith, 2015; Martin et al., 2015). Care should not be kept in an externalized, hidden spot of an ecology of practices, but rather recentralized and brought to light and at the heart of a political questioning via ‘restor[ing] some of the normally hidden aspects of network externalities’ (Star, 1990). From then on, can care for representational objects onto-politically affect the modes of relationship maintained with the ‘real objects’ that they ought to represent? In other words, can focusing on the care involved in maintaining models—while suspending the fact that they manifest a certain modern onto-epistemology—contribute to speculating about and inventing ways of relating to the world that emphasizes response-ability? If chains of transformation transport matter to form, can care for form be transposed back into matter through chains of care, which are formed and articulated by reversibly following the paths traced by reference chains? Can restorers, there in the cold basement of a museum repository, in touch with yogurt cups, acrylics, and brushes, and a responsible attention to dusty old models, teach us to take care of mountains?

In exploring this question, the case of relief models is all the more special. In the chains of reference that ground scientific representations, something remains constant through a succession of transformed and transforming inscriptions (Latour, 1999, p. 58). However, relief models mimetically resemble the mountains they re-present. In this respect, and as I have suggested, they are hybrids of art and science that appeal all the more to emotional attachments and affects. This reflects art’s unique agency, which lies in its power to fascinate and evoke various emotions; it is a ‘technology of enchantment’ (Gell, 1992, 1998). My ethnographic inquiry convinced me of this on several occasions: Among their many potentialities, relief models can arouse emotions and mediate affects. From then on—which is my speculative proposal—these attachments can be transferred to what the relief models re-present and on whose behalf they can speak, thanks to the robustness of the chains of reference, namely the mountains. Moreover, as discussed by Ballestero (2023), models have the potential to generate what she terms ‘casual planetarities’. These ‘bring into people’s everyday experiences new forms of sensing and making sense of [environmental dynamics’] geologic presence’ (Ballestero, 2023, p. 267). Models, she argues, mediate a specific ‘phenomenological accessibility’ to the environmental processes they represent through the ‘oscillations between distance and proximity’ that they can cultivate (Ballestero, 2023, p. 269). Yet, as I have shown, the care given to the relief models is also care given to a multiplicity of scales, from the tiniest fragment of wood to the vastness of the mountain made accessible by the model, and thus to the specific agency of the relief models in their capacity to generate casual planetarities.

Moreover, the care given to the relief models aligns with new conservation policies. As I mentioned earlier, these policies now embrace conservation rather than restoration, which allows for the acknowledgement of the traces of time, letting wear and cracks, caused by conflicts between different materials as well as traces of repair, remain visible. This also opens up speculative possibilities. Unlike the taxidermy pieces described by Haraway (1984–1985), which hide the stitches and thus the ‘patched-up’ nature of nature as represented, the care given to relief models reveals some ‘evidence of stitchery’ (Haraway, 1989, p. 280). When related to mountains, this opens up possibilities for imagining new forms of care that move beyond conserving an idealized nature. Instead, it envisions caring for and restoring a nature that is, in Haraway’s (1988, p. 586) words, ‘partial in all its guises, never finished, whole, simply there and original [but] always constructed and stitched together imperfectly’. While Haraway originally applied these words to ‘the knowing self’, they can symmetrically apply to nature as both an object and subject of knowledge, thus accounting for a nature that is cracked, fissured, and mended, reflecting the realities of the Anthropocene.

The question of the recursive lessons from care practices for research in the social sciences has been explored greatly and has led to fruitful proposals, such as the project to establish what Law (2021) has coined as ‘care-ful analytics’ (see also Law & Lin, 2022). In response, I ask the following questions, which I believe deserve further exploration (see Latour, 2017; Morizot, 2017; Stengers, 2010; for a discussion see Janicka, 2023). To what extent can practices of caring for representational objects recursively inform ways of fostering reciprocal and responsible relationships with the living world—relationships that these objects were originally supposed to, awkwardly and authoritatively, capture? How might such practices help dig diplomatic pathways toward the living world? Could such a hypothesis help reconcile us with scientific reference and with representational objects—objects that, having functioned as instruments of power and control, could instead be reinvested with a diplomatic mission?
